# Pharmacogenetics of OATP Transporters Reveals That *SLCO1B1* c.388A>G Variant Is Determinant of Increased Atorvastatin Response

**DOI:** 10.3390/ijms12095815

**Published:** 2011-09-09

**Authors:** Alice C. Rodrigues, Paula M. S. Perin, Sheila G. Purim, Vivian N. Silbiger, Fabiana D. V. Genvigir, Maria Alice V. Willrich, Simone S. Arazi, Andre D. Luchessi, Mario H. Hirata, Marcia M. S. Bernik, Egidio L. Dorea, Carla Santos, Andre A. Faludi, Marcelo C. Bertolami, Antonio Salas, Ana Freire, Maria V. Lareu, Christopher Phillips, Liliana Porras-Hurtado, Manuel Fondevila, Angel Carracedo, Rosario D. C. Hirata

**Affiliations:** 1 Faculty of Pharmaceutical Sciences, University of Sao Paulo, Sao Paulo 05508-900, Brazil; E-Mails: paulamsp@gmail.com (P.M.S.P.); viviansilbiger@hotmail.com (V.N.S.); fdallavecchia@yahoo.com.br (F.D.V.G.); malicewi@usp.br (M.A.V.W.); sisorkin@usp.br (S.S.A.); adluchessi@uol.com.br (A.D.L.); mhhirata@usp.br (M.H.H.); rosariohirata@usp.br (R.D.C.H.); 2 Life Technologies, Sao Paulo 04311-000, Brazil; E-Mail: sheila.purim@lifetech.com; 3 University Hospital, University of Sao Paulo, Sao Paulo 05508-000, Brazil; E-Mails: mbernik@usp.br (M.M.S.B.); egidiodr@gmail.com (E.L.D.); 4 Department of Biology, University of Aveiro, Aveiro 3810-193, Portugal; E-Mail: carla.santo@ispa.pt; 5 Forensic Genetics Unit, Institute of Legal Medicine, University of Santiago de Compostela, Galicia 15705, Spain; E-Mails: antonio.salas@usc.es (A.S.); ana.freire@usc.es (A.F.); mvictoria.lareu@usc.es (M.V.L.); christopherpaul.phillips@usc.es (C.P.); glolipo@gmail.com (L.P.-H.); manuel.fondevila@usc.es (M.F.); apimlang@usc.es (A.C.); 6 Dante Pazzanese Institute, Sao Paulo 04012-909, Brazil; E-Mail: afaludi@uol.com.br (A.A.F.); bertolami@uol.com.br (M.C.B.)

**Keywords:** OATP, atorvastatin, single nucleotide polymorphisms, pharmacogenetics

## Abstract

**Aims:**

The relationship between variants in *SLCO1B1* and *SLCO2B1* genes and lipid-lowering response to atorvastatin was investigated.

**Material and Methods:**

One-hundred-thirty-six unrelated individuals with hypercholesterolemia were selected and treated with atorvastatin (10 mg/day/4 weeks). They were genotyped with a panel of ancestry informative markers for individual African component of ancestry (ACA) estimation by SNaPshot^®^ and *SLCO1B1* (c.388A>G, c.463C>A and c.521T>C) and *SLCO2B1* (−71T>C) gene polymorphisms were identified by TaqMan^®^ Real-time PCR.

**Results:**

Subjects carrying *SLCO1B1* c.388GG genotype exhibited significantly high low-density lipoprotein (LDL) cholesterol reduction relative to c.388AA+c.388AG carriers (41 *vs.* 37%, *p* = 0.034). Haplotype analysis revealed that homozygous of SLCO1B1*15 (c.521C and c.388G) variant had similar response to statin relative to heterozygous and non-carriers. A multivariate logistic regression analysis confirmed that c.388GG genotype was associated with higher LDL cholesterol reduction in the study population (OR: 3.2, CI95%:1.3–8.0, *p* < 0.05).

**Conclusion:**

*SLCO1B1* c.388A>G polymorphism causes significant increase in atorvastatin response and may be an important marker for predicting efficacy of lipid-lowering therapy.

## 1. Introduction

Organic anion transporting polypeptides (OATPs) are plasma membrane transport proteins that mediate the active cellular influx of a variety of amphipathic compounds. OATP1B1, OATP2B1 and OATP1B3 are expressed in the sinusoidal membrane of hepatocytes and transport a large number of therapeutic drugs, such as statins [1]. The uptake of statins by OATPs not only represents the first step of hepatic drug elimination, but is also a delivery system to the liver as the target organ. Such transport therefore potentially influences the efficacy of the therapy of this drug class, as differences in OATP activity may result in variability of statins plasma levels and consequently variability in drug response.

Atorvastatin is a potent inhibitor of the 3-hydroxy-3-methlyglutaryl-coenzyme A reductase (HMGCR), the rate-limiting enzyme in the cholesterol biosynthesis pathway [2]. It plays an important role in reducing plasma low-density lipoprotein (LDL) cholesterol and in preventing the risk of coronary artery disease (CAD) [3,4]. Hepatic uptake of atorvastatin has been demonstrated to be mediated in an OATP-dependent manner [5].

OATP1B1 and 2B1 are codified by solute carrier organic anion transporter family genes, member 1B1 (*SLCO1B1*) and 2B1 (*SLCO2B1*), respectively. *SLCO1B1* has several common polymorphisms and its relation with statin efficacy remains uncertain. The single nucleotide polymorphism (SNP) *SLCO1B1* c.521T>C has been associated with markedly increased plasma concentrations of simvastatin, rosuvastatin, pravastatin, and atorvastatin [6–12]. These studies have shown that homozygous for c.521C allele presented the highest plasma concentration as compared to TC heterozygote or TT homozygote. The increase in plasma concentration of statins may increase the exposure of the drug and lead to adverse drug reactions. Indeed, SLCO1B1*5 (c.521C) was associated with increased risk of statin-induced myopathy in a genome-wide association study in patients taking simvastatin 80 mg [13]. The SNP c.388A>G (SLCO1B1*1b) has been also associated with higher activity of OATP1B1 resulting in lower oral bioavailability of pravastatin [14].

Many studies have focused only on the pharmacokinetics of statin, whereas the impact of *SLCO1B1* genotypes on lipid-lowering response to statins remains unsure. In one study, in Japanese hypercholesterolemic patients treated with pravastatin for eight weeks, heterozygous carriers of SLCO1B1*15 allele (c.388G and c.521C alleles) had poor LDL cholesterol reduction as compared with non-carriers (reduction: −14.1 *vs.* −28.9%) [15]. On the other hand, in a cohort of elderly hypercholesterolemic patients treated with fluvastatin extended-release, the *SLCO1B1* c.463C>A SNP was significantly associated with enhanced fluvastatin response [16].

The potential contribution of genetic variations in *SLCO2B1* in statins efficacy is not known. Until now, only one study has accessed the impact of variants of *SLCO2B1*; however, no differences were found [7]. OATP2B1, differently from OATP1B1 is localized not only in hepatocytes, but at membranes of enterocytes, human skeletal muscle and heart. Recent studies have suggested that OATP2B1 may play a role in statin-induced myopathy, since the presence of OATP2B1 in primary muscle myoblast cells caused a significant increase in intracellular retention of statins [17].

The uptake and delivery of atorvastatin into hepatocytes by OATP is essential for its action. Because some studies have previously associated OATP variants with altered pharmacokinetic profile of atorvastatin, the aim of this study was to describe the influence of *SLCO1B1* and *SLCO2B1* genotypes on the pharmacological efficacy of atorvastatin.

## 2. Results and Discussion

### 2.1. Characteristics of the Hypercholesterolemic Individuals

Clinical and laboratory data of the HC subjects were previously described by Rebecchi *et al.* (2009) [18]. Atorvastatin treatment significantly reduced total LDL cholesterol and triglycerides values (Table 1). Concomitant ingestion of CYP3A4 substrates or inhibitors did not affect atorvastatin response (*p* > 0.05), as evaluated by Chi-square test (data not shown). We did not observe an increase in high-density lipoprotein (HDL) cholesterol levels as it has been described for this drug. In addition, atorvastatin treatment did not cause a significant increase in CK levels. There was no report of intolerance or adverse effects related to atorvastatin therapy. We have observed an increase of ALT levels after treatment, but this increase did not translate into hepatotoxicity for the patients that have undergone atorvastatin treatment.

### 2.2. SLCO1B1 and SLCO2B1 Polymorphisms

Genotype and allele frequencies for *SLCO1B1* and *SLCO2B1* polymorphisms were calculated for this sample of the Brazilian population. As expected, allele frequencies of these variants were in Hardy-Weinberg Equilibrium confirming the random selection of the individuals. The frequencies of the three variants (c.388A>G, c.463C>A and c.521T>C) for *SLCO1B1* gene in Brazilian individuals were 32%, 16% and 12%, respectively. Minor allele frequency for *SLCO2B1* −71C allele was 53%.

Linkage disequilibrium was tested for *SLCO1B1* variants. Association was found between c.388A>G and c.521T>C polymorphisms (*D*′ = 0.84; χ^2^ = 9.56, *p* = 0.049) and c.388A>G and c.463C>A SNPs were also consistently associated (*D*′ = 1.0; χ^2^ = 69.94, *p* < 0.0001). Nevertheless, c.521T>C and c.463C>A were not associated (χ^2^ = 2.32, *p* = 0.677). Therefore, six *SLCO1B1* haplotypes were found in our study group: *1a (39.3%), *1b (33.3%), *14 (16.0%), *15 (10.3%), and *4 (1.1%).

The frequency of *SLCO1B1* and *SLCO2B1* SNPs and of their haplotypes varies largely among ethnically identified populations [19–21]. Despite the fact that the described frequencies above for *SLCO1B1* are similar to others previously reported [16,20], Brazilians are a highly admixed population with Amerindian, European and African ancestral roots and estimation of the genetic ancestry provided by AIMs may allow more realistic representations of such diversity [22–25]. For this purpose, we have estimated the ACA mean value for our sample and associated it with the alleles of *SLC* polymorphisms.

The individual ACA values across the study population ranged from 0.003 to 0.989. ACA mean values between ancestral and variant allele of each SNP are presented in Figure S1. We observed that, only for *SLCO2B1* −71T>C polymorphism, the ACA mean value was significantly higher in subjects carrying −71T allele compared to −71C allele carriers [0.461 (0.010–0.687) *vs.* 0.112 (0.037–0.243), *p* = 0.023].

Categorization of ACA values in four quartiles (<0.25; 0.25–0.50; 0.50–0.75; >0.75) revealed that frequency of the *SLCO2B1* −71C allele decreased progressively from the lowest (<0.25 ACA) to the highest (>0.75 ACA) quartile, showing its higher prevalence in people with minor African influence (Supplemental Table 1). For *SLCO1B1* gene, the frequencies of the SNPs were not different among the ACA quartiles.

*SLCO1B1* c.463C>A SNP showed a trend for decreasing the frequency of c.463A variant from low ACA values (<25%) to high ACA values (<75%) (Supplemental Figure 1, Supplemental Table 1).

These results are in agreement with previous reports showing a low prevalence of this allele in African Americans and a high prevalence in Caucasians [20]. For *SLCO2B1* variant, a significant association between −71C allele and ACA values was found. There is no study reporting this relationship, but we may conclude that −71C allele varies among ethically identified populations and presents a low frequency in people with high African background.

The variables’ age, BMI, gender, hypertension, obesity, menopause, cigarette smoking, alcohol consumption, physical activity, and baseline mean plasma lipid parameters were not different among the genotypes or haplotypes for all the polymorphisms studied (data not shown). These results suggest that *SLCO1B1* and *SLCO2B1* variants were not associated with these variables in this sample.

### 2.3. Effect of SLCO1B1 and SLCO2B1 Polymorphisms on Atorvastatin Response

Results from one-way ANOVA regarding the effect of *SLCO1B1* and *SLCO2B1* SNPs on total and LDL cholesterol are presented in Table 2. For *SLCO1B1* c.388A>G polymorphism, homozygous for c.388G allele presented higher mean percentage of LDL cholesterol reduction than carriers of c.388A allele (41.3 ± 12.4% for GG *vs.* 36.6 ± 12.1% for AA + AG, *p* = 0.034), in a dominant model. For *SLCO2B1* polymorphism there was no association between lipid parameters and the genotypes.

**Figure 1:**
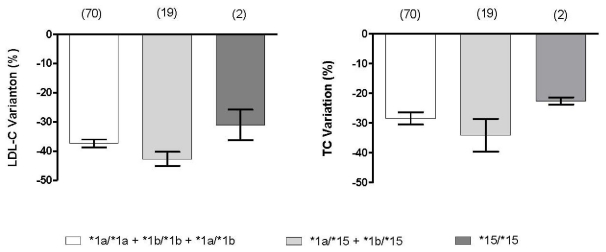
Influence of the *SLCO1B1* *15 variant on reduction of total (TC) and low-density lipoprotein (LDL-C) cholesterol in response to atorvastatin (10 mg/day/4 weeks). *P* > 0.05, as compared by One-Way Analysis of Variance followed by Hom-Sidak test. Number of individuals in parenthesis.

In addition, the effect of *SLCO1B1* haplotypes on total and LDL cholesterol before and after atorvastatin treatment was investigated. We have compared the effect of *15 homozygous (*15/*15), *15 heterozygous (*1a/*15 and *1b/*15) and *15 non-carriers. (*1a, *1b, and *1a/*1b). Despite the fact that *15/*15 subjects presented lower total and LDL cholesterol reductions than *15 heterozygous and *15 non-carriers, this association lacked statistical significance (Figure 1). There was no effect of *14 allele on atorvastatin response.

After atorvastatin treatment, LDL cholesterol serum concentrations varied largely from reduction of 61.7% to 6.4%. Therefore, individuals with LDL cholesterol in the first quartile (reduction higher than 48%) were compared with those with lower response. First, a stepwise forward multiple regression analysis including all parameters (age, BMI, gender, basal LDL cholesterol, and c.388A>G genotypes) was performed. After this analysis we concluded that BMI and gender were not related to atorvastatin response. Then, a multivariate logistic regression including all the remaining parameters was performed. Results from logistic regression showed that *SLCO1B1* c.388GG and higher LDL basal levels were the most significant factors positively related to atorvastatin response (Table 3).

*SLCO1B1* and *SLCO2B1* polymorphisms may have particularly important consequences for cholesterol-lowering therapy with HMGCR inhibitors, as OATPs (1A2, 1B1, 1B3, and 2B1) are involved in the hepatic uptake of statins [5]. Current knowledge has shown that SNPs in *SLCO1B1* may result in reduced efficacy and increased risk of systemic exposure, leading to adverse effects [5].

Studies of *SLCO1B1* SNPs have focused mainly on c.521T>C polymorphism. They have shown that c.521C allele causes reduced OATP1B1 activity, thus increasing plasma concentrations of all statins except fluvastatin [6–12]. The area under the curve (AUC) of atorvastatin was 1.5–2.0-fold higher in subjects with the 521C/C genotype than in those with the 521T/T [6,7,9].

The effect of c.521T>C polymorphism on atorvastatin therapy has been investigated in this study. We have found no association between c.521C allele carriers and changes in lipid parameters after 4 weeks of atorvastatin treatment. One reason for that lack of association may be due to a limited number of subjects with 521C/C genotype. Because only two individuals in our sample were homozygous for the variant allele they were pooled with the 521C/T genotype, then we could not effectively analyze the effect of 521C/C genotype.

Some studies characterizing the impact of *SLCO1B1* polymorphisms on lipid-lowering response have been conducted, however they mainly target pravastatin therapy [15,26–30]. Because these studies were very heterogeneous among the study population (healthy, hypercholesterolemic or elderly subjects), duration of treatment (single dose, 3 or 8 weeks, 1 year) and daily dose (20, 40 or 9.4 mg/day), divergent findings have been reported. For instance, Zhang *et al.* (2007) [30] reported an attenuated pravastatin (20 mg/day for 30 days) pharmacodynamic effect on total cholesterol in patients with 521TC heterozygous compared to 521TT homozygous. On the other hand, treatment with 40 mg pravastatin for 3 weeks caused no difference in lipid-lowering efficacy between c.521C carriers (*i.e.*, SLCO1B1*15 and *17) and non-carriers (SLCO1B1*1a).

The *SLCO1B1* c.463C>A polymorphism has been previously associated with fluvastatin response [16]. Carriers of *14 allele had better response to fluvastatin as compared to *1a/*1a or *1a/*14 genotypes. We have found no association between c.463C>A variant and atorvastatin response. In fact, this is not the first study to describe a lack of association between c.463C>A SNP and atorvastatin response. Thompson *et al.* (2005) [31] using a much larger sample (*n* = 1902) also did not find any association between this polymorphism and response to atorvastatin. The lack of effect of this polymorphism on atorvastatin response may be due to a substrate-specific effect of this OATP1B1 variant. This substrate-specific effect has been clearly shown for *SLCO1B1* c.521T>C SNP. It has been associated with a markedly reduced uptake of all statins except fluvastatin, as discussed before. Then, it is possible that *SLCO1B1* c.463C>A variant has a high affinity for fluvastatin, however it needs to be verified by transporter function analyses.

Significantly high reduction of LDL cholesterol in response to atorvastatin treatment was found in individuals homozygous for *SLCO1B1* c.388G allele when compared to c.388A allele carriers (−41.3 *vs.* −36.6%). This finding is consistent with previous *in vivo* studies reporting a higher transport function for OATP1B1 in subjects carrying *1b variant, resulting in lower oral bioavailability of pravastatin [8] and pitavastatin [32].

There is some evidence that SLCO1B1*15 variant (c.388G and c.521C) exhibits reduced transport function and play an important role in pravastatin and atorvastatin systemic exposure and elimination [6,33–35]. Lee *et al.* (2010) [35] have shown that the AUC of atorvastatin was 1.8 higher in *15/*15 subjects than in 1a/*15 and *1b/*15 and 2.2-fold than for *1a/*1a, *1a/*1b and *1b/*1b. Haplotype analysis revealed that mean percentage reduction in total and LDL cholesterol values at 4 weeks post-treatment with atorvastatin were lower in *15/*15 than in *15 heterozygous and *15 non-carries. The allele frequency of SLCO1B1*15 was 10.3% in our population, then the sample size was not enough to find many subjects carrying *15/*15 genotype, so the association lacks statistical significance. Multiple regression analysis in the study population revealed that only c.388GG was correlated with statin response.

With respect to *SLCO2B1* polymorphism we have not found significant differences between the different genotypes and atorvastatin response. A previous study also failed to find relationship between polymorphisms of *SLCO2B1* and pharmacokinetics of pravastatin [7].

## 3. Material and Methods

### 3.1. Subjects and Study Protocol

The characteristics of study design have been previously reported [18]. Briefly, 136 hypercholesterolemic (HC) individuals were selected randomly among the outpatients evaluated for the presence of risk factors for coronary artery disease (CAD) at the University Hospital of the Sao Paulo University (Sao Paulo City, Brazil). The study protocol was approved by the Ethics Committee of this institution as well the Committee of the Faculty of Pharmaceutical Sciences (University of Sao Paulo). Individuals diagnosed with thyroid, liver and kidney diseases, diabetes, and triglycerides higher than 400 mg/dL or subjects under treatment with lipid-lowering drugs, hormone replacement or oral contraceptives were not included. Pregnant women or patients with heart disease known previously were not included too.

Information on age, body mass index (BMI), gender, hypertension, obesity, menopause status, cigarette smoking, physical activity, alcohol consumption and family history of CAD were recorded.

HC patients with (LDL) cholesterol higher than 160 mg/dL, even after a low cholesterol diet during 4 weeks, were started on atorvastatin therapy, 10 mg orally once daily for 4 weeks. At the end of the protocol, the patients had a last appointment with the doctor and response to atorvastatin as well as any possible adverse reactions was evaluated. The study design was based on the recommendations of the National Cholesterol Education Program (NCEP) for treatment of high blood cholesterol in adults [36]. The dose of 10 mg atorvastatin was chosen because the patients had moderate elevations of LDL cholesterol, and LDL cholesterol goal will be achieved with low doses for these patients. In addition, the NCEP recommend checking the response to drug therapy in about 6 weeks.

Response to atorvastatin was evaluated by reduction of LDL cholesterol after the treatment, and adverse effects were monitored by measuring creatine kinase (CK) and alanine aminotransferase (ALT) enzymes.

### 3.2. Biochemical Profile and SLCO Variants Genotyping

Blood samples for biochemical profile (lipids, CK, and ALT) measurements and genomic DNA extraction were collected after an overnight fast, one day before and 4 weeks after atorvastatin treatment. All patients followed exactly the same study protocol. Laboratory methods for biochemical parameters are described elsewhere [18].

Genomic DNA was extracted from EDTA-anticoagulated blood by a salting-out procedure optimized in our laboratory [37]. Polymorphisms of *SLCO1B1* [c.521T>C (Val174Ala, rs4149056), c.388A>G (Asp130Asn, rs2306283), c.463C>A (Pro155Thr, rs11045819)], and *SLCO2B1* [−71T>C (rs2851069)] were detected by TaqMan^®^ Real time PCR. *TaqMan Drug Metabolism Genotyping Assay (20X)* were obtained from Life Technologies (Foster City, CA, USA).

PCR assays contained 4 μL of Universal Master Mix (2X) (Life Technologies), 0.4 μL of TaqMan Drug Metabolism Genotyping Assay (20X) and 3.6 μL de DNA (20 ng) diluted in nuclease-free water. The thermal cycling protocol consisted of initial cycle at 10 min a 95 °C followed by 40 cycles at 92 °C for 15 s, 60 °C for 1 min, using standard 7500 conditions. For *SLCO2B1* polymorphisms the cycles were increased to 50, and the time for extension was 90 s. The amplification was carried out in a 7500 fast real-time system (Life Technologies). Genotype calling was performed using the SDS software (Life Technologies).

### 3.3. Ancestry Informative Markers (AIMs)

The ancestral origin and African component ancestry (ACA) of the individuals was explored using a 34-plex AIM-SNPs assay. SNPs were genotyped by multiplex-PCR followed by 34-plex SNaPshot^®^ primer extension reactions (Life Technologies, Foster City, USA). Extension products were separated by capillary electrophoresis (3130 Analyzer, Life Technologies) and POP6™ polymer (details in [38]). The ACA of the samples was estimated and categorized into four ancestral categories (<0.25; 0.25–0.50; 0.50–0.75; >0.75) according to the relative contribution of a variable number of African ancestral population.

### 3.4. Statistical Analysis

For *SLCO1B1*, as previously described by Tirona and colleagues (2002) [39], haplotypes were defined based on the presence of c.388A>G, c.463C>A and c.521T>C polymorphisms alone or in combination, as follows: SLCO1B1*1a (wild type), *1b (c.388G), *4 (c.463A), *5 (c.521C), *14 (c.388G and c.463A) or *15 (c.388G and c.521C). The agreement of genotypes frequencies with Hardy-Weinberg equilibrium expectations was tested by χ^2^ test using Haploview software. Relationships between the genotypes or haplotypes and categorical variables were evaluated by the Chi-square or Exact Fisher test.

Continuous variables are presented as mean ± SD. Those without normal distribution were analyzed by a non parametric test, and they are presented as median (25%–75%). Numerical variables were compared by t test (two variables) and One-way ANOVA (three or more variables) and Holm-Sidak method was used for multiple comparisons. Logistic regression analysis was used to evaluate the relationships between reduction of serum LDL cholesterol and other variables after treatment with atorvastatin. Statistical tests were performed using the Sigma Stat version 3.5 (SPSS Inc., Chicago, IL, USA). Significance was considered *P* < 0.05.

## 4. Conclusions

The lack of association between lipid response to atorvastatin and *SLCO1B1* c.521T>C polymorphism may be due to the size of our sample since we could not find many individuals homozygous to the rare allele. This caused the statistical power of the test performed to be below the desired level. In addition, the positive association between c.388GG carriers and higher LDL cholesterol reductions would be greatly strengthened if the sample were larger.

*SLCO1B1* c.388A>G polymorphism causes significant increase in atorvastatin response and may be an important marker for predicting efficacy of lipid-lowering therapy. However, others factors, such as the drug given to the patient, duration of the treatment, daily dose, basal LDL cholesterol, may influence the efficacy of the therapy and needs to be taken into consideration.

## Supplementary Information



## Figures and Tables

**Table 1:** Biochemical profile of hypercholesterolemic individuals in response to atorvastatin (10 mg/day/4 weeks).

Variables	Basal	Atorvastatin *	Change (%)	*P*
TC (mg/dL)	281 ± 38	198 ± 30	−28.9 ± 9.5	<0.001
LDL-C (mg/dL)	193 ± 55	118 ± 27	−38.3 ± 12.4	<0.001
HDL-C (mg/dL)	56 ± 14	54 ± 13	−2.5 ± 10.5	<0.002
TG (mg/dL)	160 ± 66	132 ± 52	−26.9 ± 52.5	<0.001
CK (U/L)	102 ± 80	104 ± 88	4.9 ± 36.5	0.606
ALT (U/L)	22 ± 10	25 ± 15	23.0 ± 63.2	<0.001
ApoAI (g/L)	130 ± 25	136 ± 27	4.9 ± 15.4	0.013
ApoB (g/L)	140 ± 22	102 ± 22	−28 ± 46.1	<0.001

TC: total cholesterol, LDL-C: low-density lipoprotein cholesterol; HDL-C: high-density lipoprotein cholesterol; TG: triglyceride; CK: Creatine kinase; ALT: Alanine aminotransferase; ApoAI: Apolipoprotein AI; ApoB: Apolipoprotein B.

* 10mg/daily for 4 weeks.

**Table 2:** Association of *SLCO1B1* and *SLCO2B1* variants with total and LDL cholesterol in individuals treated with atorvastatin.

SNP	Basal		Atorvastatin		Change (%)	

	TC	LDL-C	TC	LDL-C	TC	LDL-C
***SLCO1B1***						
**c.521T>C**						
TT (108)	281 ± 37	192 ± 34	199 ± 29	118 ± 26	28.7 ± 9.1	38.1 ± 12.4
TC + CC (28)	282 ± 35	193 ± 31	192 ± 32	114 ± 28	31.8 ± 9.3	40.9 ± 11.6
*P*	0.890	0.942	0.253	0.442	0.171	0.433
**c.388A>G**						
GG (49)	279 ± 32	193 ± 36	193 ± 29	111 ± 25	30.6 ± 9.8	41.3 ± 12.4
AA + AG (82)	280 ± 40	191 ± 28	200 ± 31	121 ± 27	28.0 ± 9.2	36.6 ± 12.1
*P*	0.550	0.527	0.162	0.077	0.123	**0.034**
**c.463C>A**						
CC (95)	283 ± 38	196 ± 35	199 ± 32	120 ± 27	29.4 ± 9.3	38.4 ± 11.5
CA + AA (41)	271 ± 31	184 ± 27	194 ± 27	113 ± 26	27.9 ± 9.9	38.0 ± 14.4
*P*	0.070	0.072	0.374	0.198	0.414	0.871
***SLCO2B1***						
−**71T>C**						
TT (42)	281 ± 43	194 ± 37	200 ± 34	120 ± 28	28.6 ± 8.6	37.6 ± 10.6
TC + CC (94)	282 ± 35	198 ± 29	198 ± 29	116 ± 27	29.4 ± 9.4	39.0 ± 12.9
*P*	0.891	0.794	0.598	0.324	0.463	0.394

Number of individuals is given in parenthesis. Values are mean ± standard deviation. *P: p*-values as evaluated by one-way analysis of variance, significant *p*-values are indicated in bold. TC: total cholesterol (mg/dL); LDL-C: low-density lipoprotein cholesterol (mg/dL).

**Table 3:** Multiple logistic regression analysis for reduction of LDL cholesterol after atorvastatin treatment.

Variables	*p*-value	Odds Ratio	95% CI
Basal LDL cholesterol (≥208 mg/dL)	**0.012**	**3.47**	**1.32–9.14**
Age (<60 years)	0.077	0.45	0.19–1.09
*SLCO1B1* c.388G allele (dominant)	**0.012**	**3.23**	**1.30–8.04**

CI: Confidence interval; Significant values are highlighted in bold. LDL cholesterol reduction was considered higher than 48% of the basal level.
