# Supplementary material for: Pharmacogenetics of OATP Transporters Reveals That SLCO1B1 c.388A>G Variant Is Determinant of Increased Atorvastatin Response
Source: Int J Mol Sci. 2011 Sep 9;12(9):5815–27. doi: 10.3390/ijms12095815 (PMC3189752; doi:10.3390/ijms12095815)
Supplement: Supplementary file 1 [file ijms-12-05815-s001.pdf]

Supplementary Information

Alice C. Rodrigues [1,*], Paula M. S. Perin [1], Sheila G. Purim [2], Vivian N. Silbiger [1], Fabiana D. V. Genvigir [1], Maria Alice V. Willrich [1], Simone S. Arazi [1], Andre D. Luchessi [1], Mario H. Hirata [1], Marcia M. S. Bernik [3], Egidio L. Dorea [3], Carla Santos [4,5], Andre A. Faludi [6], Marcelo C. Bertolami [6], Antonio Salas [5], Ana Freire [5], Maria V. Lareu [5], Christopher Phillips [5], Liliana Porras-Hurtado [5], Manuel Fondevila [5], Angel Carracedo [5] and Rosario D. C. Hirata [1]

[1] Faculty of Pharmaceutical Sciences, University of Sao Paulo, Sao Paulo 05508-900, Brazil; E-Mails: paulamsp@gmail.com (P.M.S.P.); viviansilbiger@hotmail.com (V.N.S.); fdallavecchia@yahoo.com.br (F.D.V.G.); malicewi@usp.br (M.A.V.W.); sisorkin@usp.br (S.S.A.); adluchessi@uol.com.br (A.D.L.); mhhirata@usp.br (M.H.H.); rosariohirata@usp.br (R.D.C.H.)

[2] Life Technologies, Sao Paulo 04311-000, Brazil; E-Mail: sheila.purim@lifetech.com

[3] University Hospital, University of Sao Paulo, Sao Paulo 05508-000, Brazil; E-Mails: mbernik@usp.br (M.M.S.B.); egidiodr@gmail.com (E.L.D.)

[4] Department of Biology, University of Aveiro, Aveiro 3810-193, Portugal; E-Mail: carla.santo@ispa.pt

[5] Forensic Genetics Unit, Institute of Legal Medicine, University of Santiago de Compostela, Galicia 15705, Spain; E-Mails: antonio.salas@usc.es (A.S.); ana.freire@usc.es (A.F.); mvictoria.lareu@usc.es (M.V.L.); christopherpaul.phillips@usc.es (C.P.); glolipo@gmail.com (L.P.-H.); manuel.fondevila@usc.es (M.F.); apimlang@usc.es (A.C.)

[6] Dante Pazzanese Institute, Sao Paulo 04012-909, Brazil; E-Mail: afaludi@uol.com.br (A.A.F.); bertolami@uol.com.br (M.C.B.)

* Author to whom correspondence should be addressed; E-Mail: alice-rodrigues@usp.br; Tel.: +55-11-3091-3660; Fax: +55-11-3813-2197.

Received: 29 July 2011; in revised form: 29 August 2011 / Accepted: 30 August 2011 / Published: 9 September 2011

Abstract: Aims: The relationship between variants in *SLCO1B1* and *SLCO2B1* genes and lipid-lowering response to atorvastatin was investigated. Material and Methods: One-hundred-thirty-six unrelated individuals with hypercholesterolemia were selected and treated with atorvastatin (10 mg/day/4 weeks). They were genotyped with a panel of ancestry informative markers for individual African component of ancestry (ACA) estimation by SNaPshot® and *SLCO1B1* (c.388A>G, c.463C>A and c.521T>C) and *SLCO2B1* (−71T>C) gene polymorphisms were identified by TaqMan® Real-time PCR. Results: Subjects

carrying *SLCO1B1* c.388GG genotype exhibited significantly high low-density lipoprotein (LDL) cholesterol reduction relative to c.388AA+c.388AG carriers (41 *vs*. 37%, p = 0.034). Haplotype analysis revealed that homozygous of *SLCO1B1*15* (c.521C and c.388G) variant had similar response to statin relative to heterozygous and non-carriers. A multivariate logistic regression analysis confirmed that c.388GG genotype was associated with higher LDL cholesterol reduction in the study population (OR: 3.2, CI95%:1.3–8.0, $p < 0.05$). Conclusion: *SLCO1B1* c.388A>G polymorphism causes significant increase in atorvastatin response and may be an important marker for predicting efficacy of lipid-lowering therapy.

Keywords: OATP; atorvastatin; single nucleotide polymorphisms; pharmacogenetics

---

Table 1S. Frequency of the minor alleles of *SLC* polymorphisms according to ACA quartiles in the Brazilian sample.

ACA	SLCO1B1			SLCO2B1
	c.388G>A G allele	c.521T>C C allele	c.463C>A A allele	−71 T>C C allele
<0.25	0.63	0.14	0.26	0.54
0.25–0.50	0.61	0.06	0.23	0.39
0.50–0.75	0.81	0.06	0.06	0.37
>0.75	0.70	0	0	0.10
	(χ^2 = 2.311, p = 0.51)	(χ^2 = 2.421, p = 0.49)	(χ^2 = 5.823, p = 0.12)	(χ^2 = 7.9851, p = 0.046)

ACA: African component ancestry. P: p-value, as compared by Chi-Square test.

Figure 1S. Comparison of African Ancestral Component (ACA) mean values between the alleles of *SLCO1B1* and *SLCO2B1* polymorphisms. p: *p*-value, as compared by *t*-test.

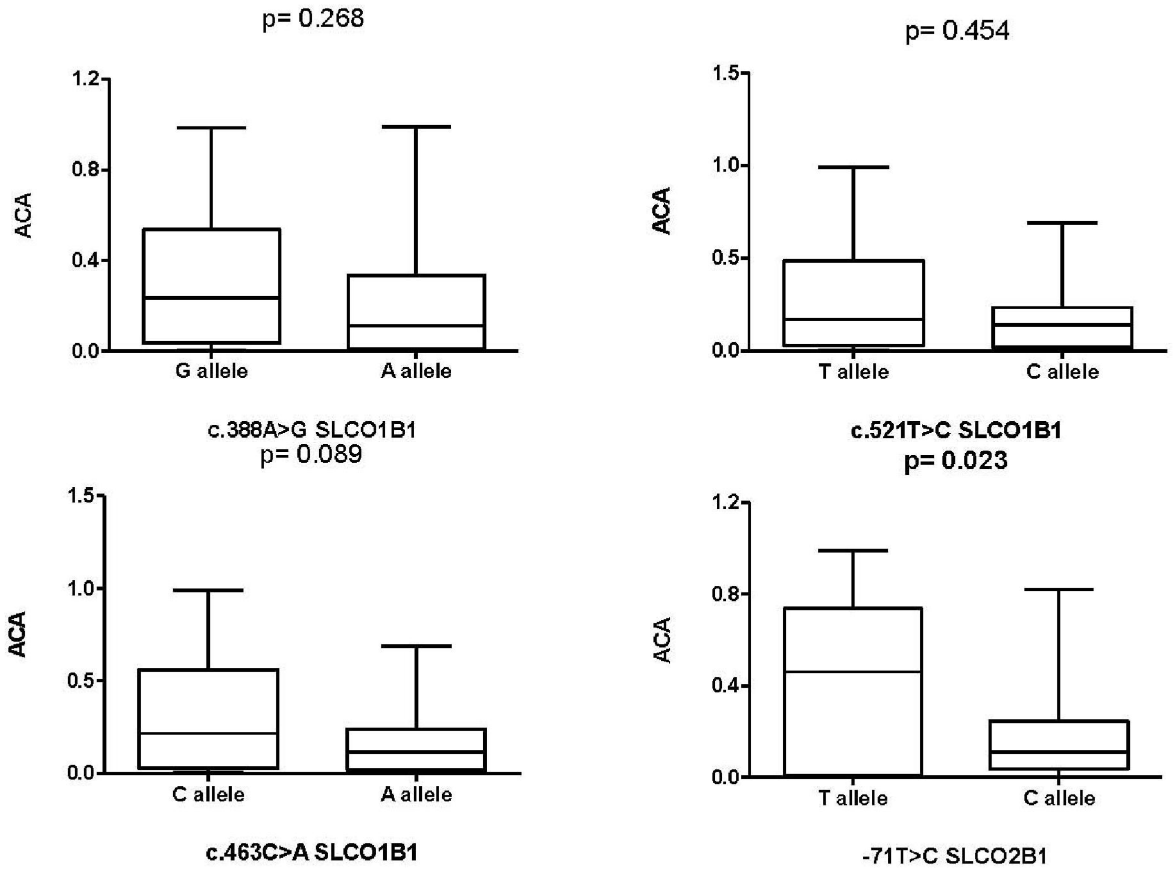

© 2011 by the authors; licensee MDPI, Basel, Switzerland. This article is an open access article distributed under the terms and conditions of the Creative Commons Attribution license (http://creativecommons.org/licenses/by/3.0/).
